# A study in Polish patients with cardiomyopathy emphasizes pathogenicity of phospholamban (*PLN*) mutations at amino acid position 9 and low penetrance of heterozygous null *PLN* mutations

**DOI:** 10.1186/s12881-015-0167-0

**Published:** 2015-04-03

**Authors:** Grażyna T Truszkowska, Zofia T Bilińska, Joanna Kosińska, Justyna Śleszycka, Małgorzata Rydzanicz, Małgorzata Sobieszczańska-Małek, Maria Franaszczyk, Maria Bilińska, Piotr Stawiński, Ewa Michalak, Łukasz A Małek, Przemysław Chmielewski, Bogna Foss-Nieradko, Marcin M Machnicki, Tomasz Stokłosa, Joanna Ponińska, Łukasz Szumowski, Jacek Grzybowski, Jerzy Piwoński, Wojciech Drygas, Tomasz Zieliński, Rafał Płoski

**Affiliations:** Laboratory of Molecular Biology, Institute of Cardiology, ul. Alpejska 42, 04-628 Warszawa, Poland; Unit for Screening Studies in Inherited Cardiovascular Diseases, Institute of Cardiology, ul. Alpejska 42, 04-628 Warszawa, Poland; Department of Medical Genetics, Warsaw Medical University, ul. Pawińskiego 3C, 02-106 Warszawa, Poland; Department of Cardiomyopathies, Institute of Cardiology, ul. Alpejska 42, 04-628 Warszawa, Poland; Department of Heart Failure and Transplantology, Institute of Cardiology, ul. Alpejska 42, 04-628 Warszawa, Poland; Department of Arrhythmia, Institute of Cardiology, ul. Alpejska 42, 04-628 Warszawa, Poland; Department of Immunology, Center for Biostructure Research, Medical University of Warsaw, Warszawa, Poland; Department of Interventional Cardiology and Angiology, Institute of Cardiology, ul. Alpejska 42, 04-628 Warszawa, Poland; Department of Epidemiology, Cardiovascular Diseases Prevention and Promotion of Health, Institute of Cardiology, ul. Niemodlińska 33, 04-635 Warszawa, Poland

**Keywords:** Dilated cardiomyopathy, Hypertrophic cardiomyopathy, Genetic testing, Phospholamban, *PLN*, Mutation

## Abstract

**Background:**

In humans mutations in the *PLN* gene, encoding phospholamban - a regulator of sarcoplasmic reticulum calcium ATPase (SERCA), cause cardiomyopathy with prevalence depending on the population. Our purpose was to identify *PLN* mutations in Polish cardiomyopathy patients.

**Methods:**

We studied 161 unrelated subjects referred for genetic testing for cardiomyopathies: 135 with dilated cardiomyopathy, 22 with hypertrophic cardiomyopathy and 4 with other cardiomyopathies. In 23 subjects multiple genes were sequenced by next generation sequencing and in all subjects *PLN* exons were analyzed by Sanger sequencing. Control group included 200 healthy subjects matched with patients for ethnicity, sex and age. Large deletions/insertions were screened by real time polymerase chain reaction.

**Results:**

We detected three different heterozygous mutations in the *PLN* gene: a novel null c.9_10insA:(p.Val4Serfs*15) variant and two missense variants: c.25C > T:(p.Arg9Cys) and c.26G > T:(p.Arg9Leu). The (p.Val4Serfs*15) variant occurred in the patient with Wolff-Parkinson-White syndrome in whom the diagnosis of cardiomyopathy was not confirmed and his mother who had concentric left ventricular remodeling but normal left ventricular mass and function. We did not detect large deletions/insertions in *PLN* in cohort studied.

**Conclusions:**

In Poland, similar to most populations, *PLN* mutations rarely cause cardiomyopathy. The 9^th^*PLN* residue is apparently a mutation hot spot whereas a single dose of c.9_10insA, and likely other null *PLN* mutations, cause the disease only with low penetrance or are not pathogenic.

**Electronic supplementary material:**

The online version of this article (doi:10.1186/s12881-015-0167-0) contains supplementary material, which is available to authorized users.

## Background

To date over 50 genes have been linked to cardiomyopathies, including *PLN* - a reversible inhibitor of cardiac sarcoplasmic reticulum calcium ATPase isoform 2a (SERCA2a). SERCA is responsible for around 70% of Ca^2+^ reuptake from cytosol into sarcoplasmatic reticulum and is a key regulator of cardiac muscle contractility [1]. PLN is a small 52 amino acid protein consisting of cytosolic domain IA (amino acids 1–20), unstructured domain IB (aa 21–30) and domain II which forms transmembrane helix. PLN is embedded in sarcoplasmic reticulum (SR) membrane close to the SERCA protein. PLN regulation of SERCA2a activity depends on PLN phosphorylation and cytosolic Ca^2+^ concentrations. PLN can be phosphorytaled at Serine 16 by the cAMP-dependent protein kinase (PKA) and at Thyrosine 17 by Ca^2+^ - calmodulin-dependent protein kinase (CAMKII) [2] or serine/threonine kinase Akt, also referred to as protein kinase B [3]. In the dephosphorylated state PLN binds to SERCA2a decreasing its affinity for Ca^2+^. The monomer form of PLN inhibits SERCA2a calcium re-uptake from cytosol into the SR [4] whereas phosphorylation by PKA enhances the formation of PLN pentamers and relieves the inhibition of SERCA2a. Mutations in *PLN* have been found in dilated cardiomyopathy (DCM), arrhythmogenic right ventricular cardiomyopathy (ARVC) and hypertrophic cardiomyopathy (HCM) patients [5-7]. So far only one c.T116G:(p.Leu39Ter) truncating PLN mutation has been described [8]. The prevalence of *PLN* mutations in DCM patients is generally below 1.5%, but in Dutch population the c.40_42delAGA:(p.Arg14del) mutation was found in 12% of DCM and 15% of ARVC patients [5,6]. As this has not been studied before, the aim of our project was to determine the prevalence of *PLN* mutations in Polish cardiomyopathy patients.

## Methods

### Patients

The study cohort was drawn from all index patients referred for clinical and genetic testing for cardiomyopathies from 1^st^ January, 2010 to 31^st^ March 2014 to the Unit for Screening Studies in Inherited Cardiovascular Diseases. It consisted of 161 unrelated probands (30.4% females, mean age 40.9 year, standard deviation 12.9), 135 with DCM, including 20 heart transplant recipients, 22 with HCM and 4 with a diagnosis of other cardiomyopathies (three with left ventricular non-compaction (LVNC) and one with restrictive cardiomyopathy (RCM)). The diagnosis was based on ESC criteria [9] after a careful examination of medical records and clinical work-up including standard 12-lead electrocardiogram (ECG) and two-dimensional Doppler echocardiography in all probands except for heart transplant recipients, whose medical records were reviewed retrospectively. Further diagnostic tests - 24-hour Holter ECG monitoring, exercise stress test were performed if necessary. Creatine phosphokinase (CPK) concentration was obtained whenever possible. All DCM probands underwent either coronary angiography or, more recently, coronary computed tomography angiography (CTA). Cardiac magnetic resonance (CMR) imaging was performed in selected patients at the discretion of the referring physician. Cardiomyopathy was considered familial when two subjects in a family met the diagnostic criteria for the same cardiomyopathy as proband’s.

### Controls

Age and sex matched control group (N = 200, 30% females, mean age 40.5 years with SD 13.0) was selected from WOBASZ II study, which is a cross-sectional population-based study aimed at delineation of classical and genetic risk factors for cardiovascular diseases. The WOBASZ II participants were randomly chosen from the Polish population register of permanent residents aged 20–74 years from the whole territory of Poland [10]. Each patient and their relative(s) gave their written informed consent to participate in the study in accordance with the Declaration of Helsinki and the study protocol was approved by the local bioethics committee (Ethics Committee of the Cardinal Wyszynski Institute of Cardiology).

### Genetic testing

DNA was extracted from the peripheral blood by phenol extraction. Multiple gene screening was initially performed by next-generation sequencing (NGS) in 23 patients (in 15 patients whole exome sequencing (WES) was performed; the remaining eight subjects were tested by targeted sequencing of a panel of 35 genes involved in cardiomyopathies, Additional file 1). WES was performed on HiSeq 1500 using TruSeq Exome Enrichment Kit (Illumina) as described previously [11]. Targeted sequencing of 35 genes was performed using a custom design SeqCap EZ Choice Library (Roche NimbleGen) of ~0,34 Mb target genomic sequences. The whole procedure was carried out according to SeqCap EZ Library SR User’s Guide v.3.0, with modifications, briefly described below. 1 μg of genomic DNA was fragmented by a 2 min sonication using Covaris M220. Genomic libraries were prepared using TruSeq Library Preparation Kit (Illumina), according to the low-throughput protocol (revision C, June 2011) with gel-free size selection method employing Agencourt AMPure XP (Beckman Coulter) magnetic beads. Sample libraries were pooled (12-plex) to a total of 1 μg and hybridized with SeqCap EZ probes for 3 days at 47°C. Captured sequences were purified with Dynabeads M-270 Streptavidin (Invitrogen), amplified, cleaned up and proceeded to sequencing. During the procedure, sample libraries and post-capture libraries were amplified using Kapa HotStart ReadyMix (Kapa Biosystems) instead of the recommended PCR master mix and cleaned up using AMPure XP beads. Libraries were quantified fluorometrically using Qubit 2.0 (Life Technologies) and quality assessment was done on 2100 Bioanalyser (Agilent Technologies). The libraries were sequenced on HiSeq 1500 (Illumina).

Prompted by finding of a mutation in the *PLN* gene c.26G>T:(p.Arg9Leu) in one patient studied by WES we performed single-gene testing of *PLN* by Sanger sequencing in additional 138 cardiomyopathy patients as well as the 23 subjects initially studied by NGS (161 subjects in total). Primers were designed to include the promoter region (up to −316 bp upstream of the transcript start site) with the first (non-coding) exon, the second (protein coding) exon and the splice sites (Additional file 2). In the control group Sanger sequencing was limited to *PLN* exon 2 (the only protein coding *PLN* exon). The PCR conditions were: 5 min of initial denaturation at 95°C, followed by 32 cycles of 30 sec at 95°C, 55 sec at 62°C, 1 min at 72°C and final extension of 10 min at 72°C. PCR products were examined on 2 % agarose gels, cleaned-up with exonuclease I and alkaline phosphatase (Thermo Scientific) and sequenced using a 3500xL Genetic Analyzer (Applied Biosystems) and BigDye Terminator v3.1 Cycle Sequencing Kit (Applied Biosystems) according to the manufacturer’s instructions. The results were analyzed with Variant Reporter 1.1 Software (Applied Biosystems).

Among the patients we also performed an analysis of copy number variations (CNV) in *PLN* by real-time PCR (RT-PCR) using 7500 Real-Time PCR Systems (Applied Biosystems). Reactions were run in duplicates in 15 μl reaction volume including MESA GREEN MasterMix Plus, Low ROX (Eurogentec, Belgium), with final primer concentrations in reaction: forward 133 nM, reverse 400 nM and 20 ng of DNA. Albumin (*ALB*) was used as a reference gene. In each assay control DNA and no-template control were included. The PCR conditions were: 10 min at 95°C, followed by 40 cycles of denaturation at 95°C for 15 sec and annealing and elongation step at 60°C for 1 min. Each amplification stage was followed by dissociation stage: 95°C for 15 sec, 60°C for 1 min. Data were analyzed using the 7500 analysis software (version 1.2.3) and calculated using ΔΔCt method.

## Results

We indentified three heterozygous variants in exon 2 of *PLN* [NM_002667.3]: a novel c.9_10insA:(p.Val4Serfs*15) and two previously described: c.25C > T:(p.Arg9Cys) (rs111033559) and c.26G > T:(p.Arg9Leu). Arg9Leu was found by WES and the other two variants by Sanger sequencing. All variants were confirmed by Sanger sequencing of two DNA samples from the same individual, including a sample isolated from independently collected blood. Neither these nor any other protein coding *PLN* variants were found in age and sex matched control group of 200 individuals.

### PLN c.9_10insA:(p.Val4Serfs*15)

**Figure 1:**
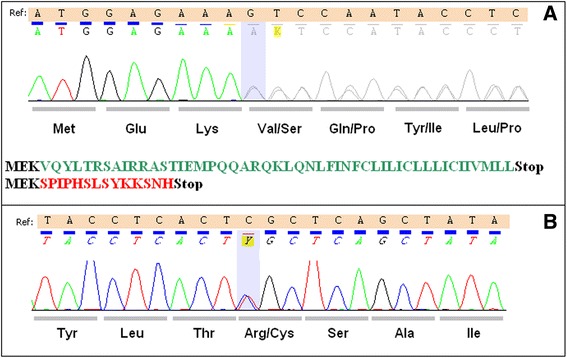
**Chromatograms of** ***PLN*** **c.9**_10insA:(p.Val4Serfs*15) and c.25C > T:p.Arg9Cys variants. Chromatograms from direct sequencing by the Sanger method showing the c.9_10insA:(p.Val4Serfs*15) **(A)** and c.25C > T:p.Arg9Cys **(B)** variants. Below chromatogram A predicted effect of c.9_10insA:(p.Val4Serfs*15) variant on *PLN* protein sequence. Black font color denotes unaffected amino acid residues, green font original *PLN* amino acid sequence, red font altered amino acid residues of *PLN* in c.9_10insA:(p.Val4Serfs*15) variant.

The novel *PLN*:c.9_10insA:(p.Val4Serfs*15) variant was identified in a 31-year-old male patient treated with radiofrequency ablation for Wolf-Parkinson-White (WPW) syndrome. He was initially diagnosed with a suspicion of LVNC but mild hypertrabeculation in the LV apex did not meet the criteria for LVNC. His left ventricular size and function were normal (LVEF = 62%). Ulcerative colitis was a coexistent disease. His both parents were asymptomatic, and normal in 12-lead standard electrocardiogram and two-dimensional Doppler echocardiogram. His 51-year-old mother, also the variant carrier, was found to have high normal left ventricular ejection fraction of 76% and concentric left ventricular remodelling, left ventricular mass was normal (72 g/m^2^). Chromatogram illustrating *PLN* c.9_10insA:(p.Val4Serfs*15) variant is shown in Figure 1A.

### PLN c.25C > T:(p.Arg9Cys)

The c.25C > T:(p.Arg9Cys) mutation was identified in a 30-year-old female who had had acute onset of DCM following viral-like syndrome at the age of 21 years with subsequent exacerbations of heart failure not responding to standard treatment and leading to heart transplant at the age of 22 years. There was no DCM or sudden cardiac deaths in the family. Her parents and two siblings are asymptomatic and refused genetic testing. Chromatogram illustrating Arg9Cys mutation is shown in Figure 1B.

### PLN c.26G > T:(p.Arg9Leu)

**Figure 2:**
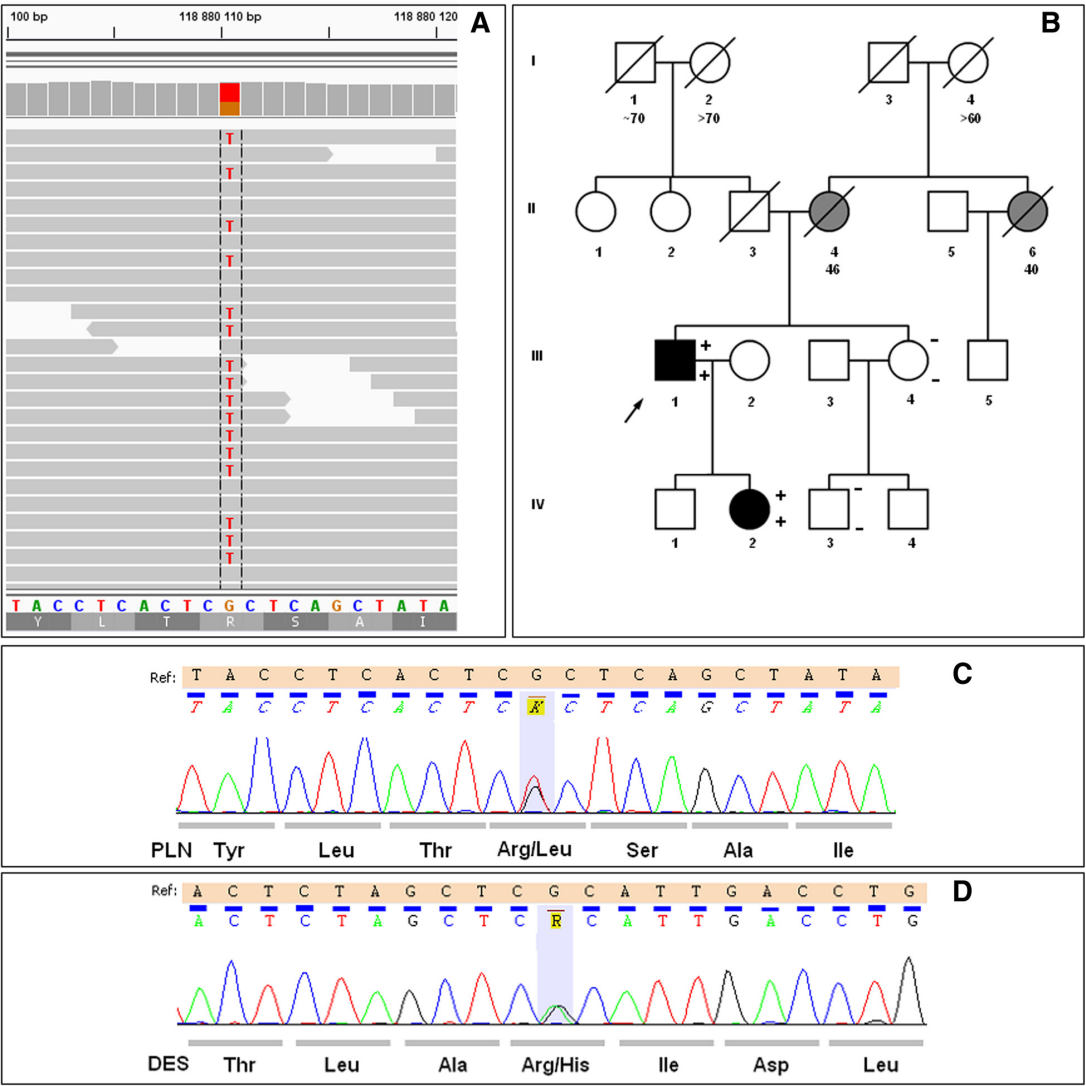
***PLN*** **c.26G > T :p.Arg9Leu and** ***DES*** **:c.665G > A:(p.Arg222His) variants IGV view of** ***PLN*** **c.26G > T :p.Arg9Leu variant found by whole exome seguencing (A), pedigree of the family (B) and chromatograms from direct sequencing by the Sanger method showing the** ***PLN*** **p.Arg9Leu variant (C) and** ***DES*** **Arg222His variant (D) in the proband.** Pedigree: squares represent males and circles represent females. An arrowhead denotes the proband. A diagonal line marks deceased individuals. Solid black symbols denote dilated cardiomyopathy and grey shading sudden death. Open symbols with asterisk denote unaffected individuals. The presence or absence of a mutation is indicated by a +/− symbol, respectively (top *PLN* Arg9Leu variant, bottom *DES* Arg222His variant).

**Figure 3:**
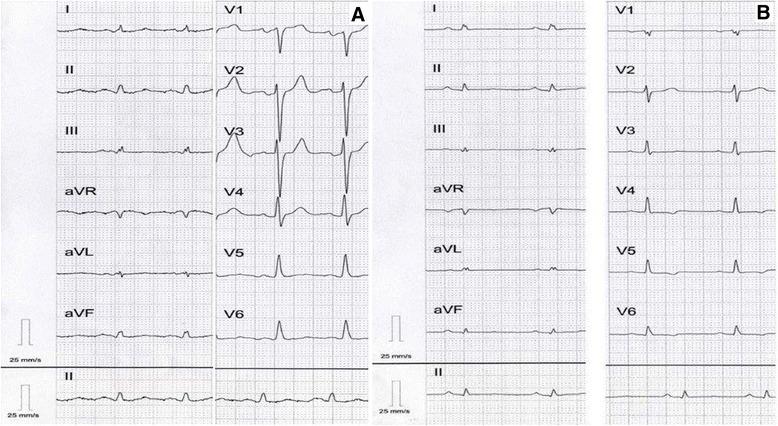
**Standard 12-lead electrocardiogram in the proband (A) and his daughter (B).** Regular sinus rhythm in both subjects, low QRS voltage in limb leads **(A)**, and in all leads **(B)**. In addition, in the proband **(A)** ST-T changes in inferolateral leads as well as left atrial enlargement. Diffuse ST-T changes were identified in the probands’s daughter **(B)**.

**Figure 4:**
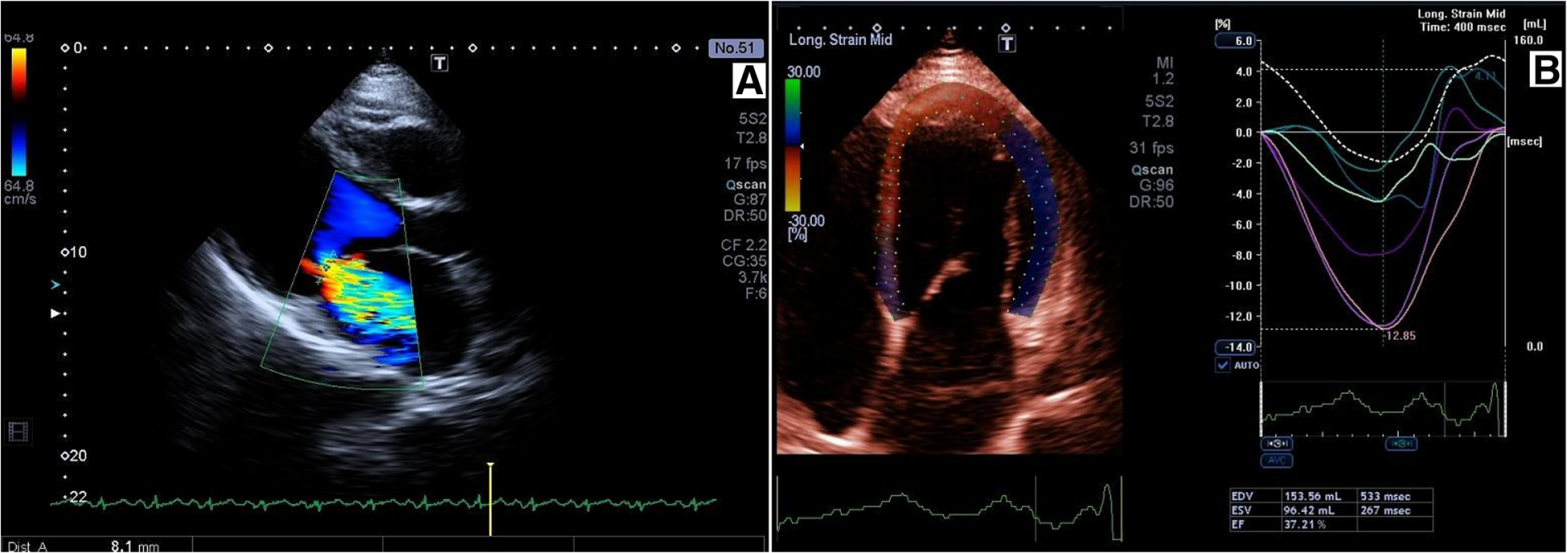
**Two-dimensional echocardiographic study of proband III-1. A**: Parasternal long axis view in systole with color flow Doppler. Severe mitral valve regurgitation (vena contracta of 8 mm) due to restriction of the mitral valve leaflets. **B**: Apical four-chamber view, speckle tracking method. Enlarged left ventricle, left ventricular end-diastolic volume (LVEDV 154 ml) with low ejection fraction (LVEF 37%). Enlarged left atrium chamber.

**Figure 5:**
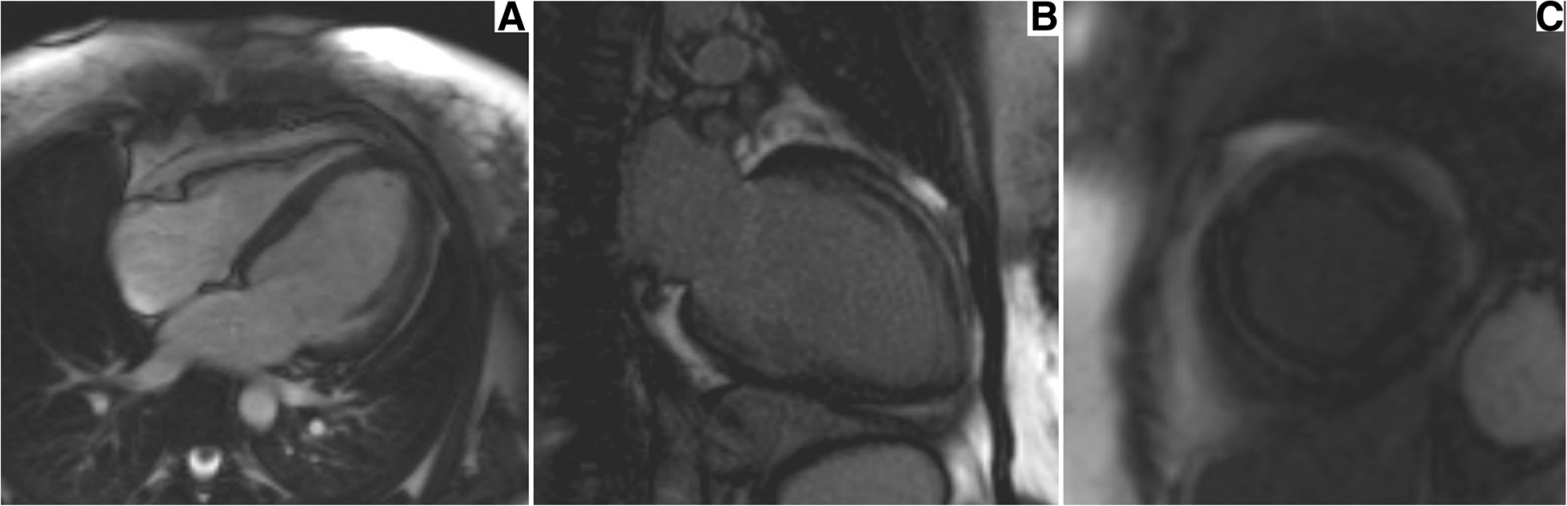
**Cardiac magnetic resonance cine image of proband’s daughter IV-2, 4-chamber image (A) demonstrating dilated left ventricle in end-diastole, late gadolinium enhancement images in 2-chamber view (B) and mid-ventricular short axis view (C) showing mid wall enhancement of the anterior and inferior wall (B) and interventricular septum (C).**

In WES mean coverage of DNA sample with the Arg9Leu mutation was 62-fold with 83.54% of the targeted exons covered at least 10× and 78.8% covered at least 20×. The total number of variants in the proband was 64754, of which 3813 were considered rare (frequency in the database from NHLBI GO exome sequencing project (ESP) [12] less-than or equal to 0.001 and frequency in our in-house database of 200 exomes less or equal to 0.01. Further filtering for variants in genes linked with DCM from Human Gene Mutation Database (HGMD) [13] revealed 48 variants, of which two were located in exons: *PLN*: c.26G > T:(p.Arg9Leu) in phosholamban and *DES*:c.665G > A:(p.Arg222His) (rs367961979) in desmin. Both variants were found in the proband III-1 and his daughter IV-2 with DCM and absent in two healthy family members III-4 and IV-3 (Figure 2). The proband at the time of genetic inquest was 55 years old, DCM was diagnosed at the age of 51. At the onset of the disease pulmonary edema in the course of atrial flutter (165/min) was present with dilated left ventricle 6.7 cm, decreased LVEF 30% and moderate mitral insufficiency on echocardiography. On intravenous amiodarone the patient returned to sinus rhythm. Additionally, on 12-lead ECG low voltage QRS complexes were found in the limb leads with QRS width of 104 ms (Figure 3A). Coronary angiography was normal. During a 4-year follow-up progression of heart failure symptoms, paroxysmal atrial fibrillation and ventricular arrhythmia 1900Vex/24 h with 5 episodes of nonsustained ventricular tachycardia (nsVT) (4 beats) occurred despite standard heart failure treatment plus amiodarone. Severe mitral valve regurgitation (vena contracta of 8 mm) due to restriction of the mitral valve leaflets was present (Figure 4). The patient refused to undergo either mitral valve insufficiency correction or implantable cardioverter-defibrilator (ICD) therapy. NT-proBNP and troponin I serum concentrations were constantly elevated (914.30 pg/mL and 0.136 ng/mL, respectively). The only comorbidity was hyperlipidemia. The first manifestations of the disease in the proband’s daughter were fatigue and frequent palpitations at 30 years of age. There were no abnormalities on physical examination at admission and no cardiovascular risk factors. Low voltage QRS complexes in all leads and ST-T changes in leads V4-V6 were found (Figure 3B). All laboratory tests were normal with the exception of NT-proBNP, whose concentration was mildly elevated (586 pg/mL). On 24 h-Holter ECG monitoring frequent ventricular ectopy 3430/24 h with 3 episodes of nsVT (3–6 beats) was observed. Echocardiogram revealed dilated left ventricle 60 mm, %LVE 128.3 global hypokinesis of the left ventricle with LVEF of 35%, slight mitral insufficiency, normal RV function and non-dilated atria. CMR showed increased left ventricular volume and marked hypokinesis of the left ventricle [LVEF of 33%, LVEDV/BSA 135 ml/m^2^ (N: 62–96), normal LV mass 60 g/m^2^ (N:47–77) (Figure 5)]. Contrast enhanced CMR imaging showed diffuse areas of midwall late gadolinium enhancement in the anterior wall and interventricular septum, which are typical of non-ischemic origin and may be observed in various diseases such as DCM, myocarditis or HCM [14]. The patient received standard heart failure treatment. During a two-year follow-up while on metoprolol tartate 100 mg and ramipril 7.5 mg, the number of ventricular arrhythmia episodes increased 10191 Vex/24 h, 34nsVT, 540 couplets while in sinus rhythm 45–116, mean 73 bpm. The patient had clinical indications to ICD implantation, however she did not agree. The proband’s mother and the mother’s sister died suddenly at age 46 and 40, respectively.

In 19/161 patients the *PLN*:c.-36 A > C (rs77186188) promoter variant was found (heterozygous in 18 patients and one homozygous, allelic frequency 6.21%). In another DCM patient heterozygous 5′UTR variant *PLN*:c.-56C > T (rs189611501) was found.

## Discussion

While screening 161 Polish patients referred for clinical cardiomyopathy diagnosis for *PLN* single nucleotide variation (SNV) we have found three heterozygous variants of note: a novel c.9_10insA:(p.Val4Serfs*15) and two previously described variants - c.25C > T:(p.Arg9Cys) and c.26G > T:(p.Arg9Leu). In the controls there were no variants in the *PLN* coding sequence.

The novel c.9_10insA:(p.Val4Serfs*15) variant was found in a patient with WPW syndrome with suspected but eventually not confirmed diagnosis of LVNC and his mother with normal LVEF and concentric left ventricular remodeling. The c.9_10insA is predicted to encode a 17 amino acid protein with only 3 initial residues corresponding to *PLN* sequence (Figure 1A). Thus, the c.9_10insA mutation can be regarded as a null mutation similar to complete deletion, which so far has not been described in humans. The only described variant leading to a truncated PLN protein is c.116A > C:p.Leu39Ter (rs111033560) [8]. Although no clear function of *PLN* Leu39Ter could be demonstrated, the truncated protein was detected in transfected human embryonic kidney (HEK) 293 cells raising a possibility that it may be expressed and exert pathologic effects and thus should not be regarded as a *bona fide* null. Whereas pathogenicity of p.Leu39Ter homozygosity is supported by two siblings who both had DCM and HTx [8], the effect of a single dose of p.Leu39Ter is less clear. Once the probands were excluded, there were a total of 16 described family members with Leu39Ter mutation from whom only two were clearly affected (DCM and HCM, respectively), four had left ventricular hypertrophy and ten were asymptomatic [7,8]. However, the subject with HCM was a 3-year-old child who inherited Leu39Ter mutation from her healthy 38-year-old mother raising a possibility that the *PLN* defect was not casually linked with HCM in this family [7]. Single dose of Leu39Ter has also been found in a 57-year-old female with a normal ECG, thickened aortic valves, moderate aortic regurgitation but normal LVEF (60%) from ClinVar project [15]. Thus, our results and the data reported in the literature indicate that in humans heterozygous null *PLN* mutations may have low penetrance or maybe are not pathogenic. This is a clinically important issue as it emphasizes that in dominant disorders null gene variants should not be presumed to be pathogenic without additional evidence.

*PLN* Arg9Cys mutation found by us in a 30-year-old heart transplantation recipient has been previously described in a large family with DCM with a similarly aggressive disease course as in our patient [16]. No history of DCM in our patient’s family suggests a *de novo* mutation, however we could not confirm this since her relatives refused genetic testing. In our study the Arg9Leu mutation was found in a familial DCM case in 55-year-old proband and his 30-year-old daughter. In addition they both carried the c.665G > A:(p.Arg222His) (rs367961979) variant in *DES* which was absent in two other healthy family members. Allele frequency of this variant is 0.0233% (two heterozygotes among 4300 participants of ESP project) [12]. rs367961979 affects conserved amino acid and is localized in helical 1B domain of desmin protein. SIFT and PolyPhen2 prediction scores indicate that it is damaging and possibly damaging, respectively. Although it is intriguing that ~50 % of patients with mutation in *DES* have cardiomyopathy, most commonly DCM [17], at present we classify *DES* c.665G > A (p.Arg222His) as a variant of unknown significance (VUS).

Similarly to what was seen in two unrelated female Arg9Leu mutation carriers studied by Medeiros et al. [5] substantial clinical differences were observed between the two Arg9Leu mutation carriers from the family studied by us. The proband’s daughter had the onset of the disease 2 decades earlier than her father, no atrial arrhythmia, low QRS voltage in all leads (proband in limb leads only) and no mitral insufficiency. It is possible that clinical manifestations of the disease are linked to genetic background or sex. In humans basal *PLN* Ser16 phosphorylation in males is higher than in females [18], thus it is possible that mutations influencing Ser16 phosphorylation have higher impact on females.

The 9^th^ amino acid of *PLN* is an apparent hot spot for mutation as the arginine at this position has also been reported to mutate to His in addition to Cys or Leu [5,16]. Functional studies indicate that possible mechanism of detrimental effect of the Arg9Leu and Arg9Cys mutations on PLN protein function is associated with impaired phosphorylation by PKA and impaired SERCA activity [16,19,20]. Mice overexpressing Arg9Cys transgene under the control of cardiac α-myosin heavy chain promoter had developed DCM and heart failure phenotype [16]. Recently a detailed mechanism of deleterious effect of Arg9Cys mutation on SERCA function was proposed [21]. Arg9Cys mutation stabilizes PLN pentamers by disulphide bridges formation and thus prevents phosphorylation of PLN by PKA and formation of PLN monomers. Disulfide bonds are more stable under oxidative stress conditions which are frequent in such a pathological states as heart failure [22]. Furthermore, under oxidative conditions the Arg9Cys mutant *PLN* can form dimers also with WT *PLN*, which were proposed to be defective in regulating SERCA activity [21].

Based on a report on a large 624 kb duplication including the *PLN* gene in a childhood-onset DCM [23] case we screened for *PLN* gene CNV but did not detect any. *PLN* promoter variant −36 A > C (rs77186188) was observed with similar allelic frequency as in 1000 genomes project (6.21% vs. 7%, respectively). Initially −36 A > C variant was reported as associated with heart failure, however further studies on larger groups of subjects did not confirm this finding [24,25]. In one DCM patient a rare 5′UTR variant (*PLN*:c.-56C > T, rs189611501) was found. This variant has an allelic frequency of 0.0014 (3 carriers per 1092 subjects) and probably is a rare polymorphism.

## Conclusions

In conclusion, our study supports the pathogenicity of *PLN* Arg9Cys and Arg9Leu variants, thus emphasizing the importance of phospholamban amino acid position 9. The phenotype of two c.9_10insA:(p.Val4Serfs*15) variant carriers suggests that heterozygous truncating *PLN* mutations have low penetrance or do not cause cardiomyopathy at all.

## Additional files

Additional file 1: Table S1. List of genes in cardiomyopathy panel. Additional file 2: Table S2. Sequences of primer used in the study.

## Abbreviations

DCM: Dilated cardiomyopathy
HCM: Hypertrophic cardiomyopathy
ICD: Implantable cardioverter-defibrilator
LVEF: Left ventricular ejection fraction
nsVT: Nonsustained ventricular tachycardia
LVNC: Left ventricular non-compaction
SNV: Single nucleotide variation
VUS: Variant of unknown significance
WPW: Wolf-Parkinson-White
